# FOXM1a Isoform of Oncogene FOXM1 Is a Tumor Suppressor Suppressed by hnRNP C in Oral Squamous Cell Carcinoma

**DOI:** 10.3390/biom13091331

**Published:** 2023-08-30

**Authors:** Rong Jia, Xiaoxuan Che, Jun Jia, Jihua Guo

**Affiliations:** 1 State Key Laboratory of Oral & Maxillofacial Reconstruction and Regeneration, Key Laboratory of Oral Biomedicine Ministry of Education, Hubei Key Laboratory of Stomatology, School & Hospital of Stomatology, Wuhan University, Wuhan 430072, China; jiarong@whu.edu.cn (R.J.); 2015203040033@whu.edu.cn (X.C.); 2 Department of Oral and Maxillofacial Surgery, School & Hospital of Stomatology, Wuhan University, Wuhan 430072, China; 3 Department of Endodontics, School & Hospital of Stomatology, Wuhan University, Wuhan 430072, China

**Keywords:** FOXM1, hnRNP C, alternative splicing, oral squamous cell carcinoma

## Abstract

FOXM1 is an oncogenic transcriptional factor and includes several isoforms generated by alternative splicing. Inclusion of alternative exon 9 produces FOXM1a, a transcriptionally inactive isoform. However, the role of FOXM1a in tumorigenesis remains unknown. In addition, the regulatory mechanisms of exon 9 splicing are also unclear. In the present study, we found that overexpression of FOXM1a significantly reduced cell proliferation and colony formation of oral squamous cell carcinoma (OSCC) cell proliferation in vitro. Importantly, OSCC cells with FOXM1a overexpression showed significantly slower tumor formation in nude mice. Moreover, we identified a U-rich exonic splicing suppressor (ESS) which is responsible for exon 9 skipping. Splicing factor heterogeneous nuclear ribonucleoprotein C (hnRNP C) can bind to the ESS and suppress exon 9 inclusion and FOXM1a expression. Silence of hnRNP C also significantly suppresses OSCC cell proliferation. HnRNP C is significantly co-expressed with FOXM1 in cancers. Our study uncovered a novel regulatory mechanism of oncogene FOXM1 expression in OSCC.

## 1. Introduction

FOXM1 (also known as MPP2, TGT3, HFH11, HNF-3, INS-1, TRIDENT, and MPHOSPH2) belongs to the Forkhead box protein family which contains a conserved winged helix DNA binding domain [1]. It is an important proliferation-associated transcriptional factor and expressed in all dividing cells. Deletion of FOXM1 is embryonically lethal. FOXM1 has been implicated as an oncogene and regulates multiple aspects of oncogenesis, including tumor cell growth [2], epithelial-to-mesenchymal transition (EMT) [3], angiogenesis [4], metastasis [5], drug resistance [6], immunosuppression [7], and so on. The human FOXM1 gene has ten exons. Exon 6 and exon 9 are alternative exons. Therefore, FOXM1 has at least four isoforms generated by alternative splicing [8]. Two isoforms without exon 9, FOXM1b and FOXM1c, are transcriptional activators and oncogenic. FOXM1a contains all exons, including exon 9, and encodes the longest FOXM1 protein. In contrast to FOXM1b and FOXM1c, FOXM1a is transcriptionally inactive [9]. However, the function of FOXM1a in tumorigenesis remains unknown. In addition, the regulatory mechanisms of exon 9 splicing are also unclear.

Alternative splicing (AS) of pre-mRNA is an important post-transcriptional regulatory mechanism in eukaryotic organisms. More than 90% of human genes are alternatively spliced [10]. Alternative splicing dramatically increases the coding capacity of the human genome. For example, the *CD44* gene can encode multiple isoforms, ranging in size from 85 kDa to 250 kDa [11]. AS often leads to the generation of isoforms with different or even opposite functions. Therefore, aberrant AS of pre-mRNA has been increasingly attracting attention as a key feature of cancer [12,13]. Cancers often take advantage of aberrant alternative splicing to produce oncogenic isoforms and inactivate tumor suppressive isoforms. It is essential to understand the regulatory mechanisms of tumorigenesis-associated alternative splicing, which may help develop novel anti-tumor therapeutic method by correcting aberrant AS.

Oral squamous cell carcinoma (OSCC) is the most common type of oral cancer and is the sixth most prevalent cancer worldwide [14]. Despite the progress in the treatment of OSCC, its mortality rates remain high [15]. There is still a lack of effective therapies for OSCC patients with advanced stages. Therefore, it is urgent to explore the underlying mechanisms of OSCC and develop new treatment methods. We and other groups have demonstrated that aberrant AS of some genes was closely associated with the tumorigenesis of OSCC, such as STAT3 [16], HNRNPL [17], SRSF3 [18], MCL1 [19], survivin [20], USO1 [21], ORAOV1 [22], and so on. However, the role of AS in OSCC need further exploration.

In the present study, we explored the roles of FOXM1a in oral squamous cell carcinoma in vitro and in vivo. Moreover, we explored the key regulatory sequences in exon 9 and identified an exonic splicing suppressor (ESS) in exon 9, which is responsible for exon 9 skipping. The splicing factor interacting with this ESS was identified by RNA-pulldown assay. The function of associated splicing factor hnRNP C in cellular proliferation of OSCC cell lines was evaluated. Our study uncovered a novel regulatory mechanism of FOXM1 expression, which suggests that blocking the interaction between hnRNP C and the ESS in exon 9 may be a novel anticancer method.

## 2. Materials and Methods

### 2.1. Cells

CAL 27 [an OSCC cell line, from China Center for Type Culture Collection (CCTCC), China], HEK 293 (Cellcook, Guangzhou, China) cells were grown in Dulbecco’s modified Eagle medium (DMEM, HyClone, Logan, UT, USA) with 10% fetal bovine serum (FBS, Thermo Fisher Scientific, Waltham, MA, USA) and 1% antibiotic-antimycotic. OSCC cell line SCC-9 cells were cultured in DMEM/F12 with 10% FBS, 1% antibiotic-antimycotic, and 400 ng/mL hydrocortisone.

### 2.2. Plasmids

The human FOXM1a, FOXM1b, or FOXM1c gene was obtained from CAL 27 cells by RT-PCR with primers 5′-ATGAAAACTAGCCCCCGTCG-3′ and 5′-CTACTGTAGCTCAGGAATAAACTGGGAC-3′. A T7-tag was fused with the 5′ end of FOXM1a, FOXM1b, or FOXM1c gene by overlapping PCR. The fused T7-FOXM1a, T7-FOXM1b, or T7-FOXM1c fragment was then subcloned into pLVX-IRES-Puro at EcoRI and NotI sites to produce T7-tagged FOXM1a, FOXM1b, or FOXM1c expression plasmid.

FOXM1 exon 9 splicing minigene was constructed as follows: human FOXM1 genomic sequence from 3′ part of exon 8 to 5′ part of exon 10 was amplified from CAL 27 cells by primer 5′-GGAAGATGAAGCCACTGCTACCACG-3′ and 5′-CAGATCCACTTGTCTGGGTCCCTG-3′, and then cloned into pEGFP-N1. Serial mutation of minigene plasmids was constructed by overlapping PCR with corresponding primers (Table 1). 

### 2.3. RNAi

The anti-human hnRNP C siRNAs were synthesized in GenePharma (Shanghai, China). The sequences of anti-hnRNP C siRNAs are 5′-CAACGGGACUAUUAUGAUA-3′ and 5′-GAUGAAGAAUGAUAAGUCA-3′, respectively. Non-specific (NS) siRNA was obtained from GenePharma. CAL 27, or SCC-9 cells were inoculated into a 6 well-plate and transfected with 20 nM hnRNP C or NS siRNA in the presence of Lipofectamine 2000 (Invitrogen, Waltham, MA, USA). After two days, the cells were passed and transfected again. After four days, the number of cells was counted. The total cellular protein was collected by 2 × SDS sample buffer and denatured by boiling for 5 min. The total RNA was purified by AxyPrep multisource total RNA miniprep kit (Axygen, Hangzhou, China).

### 2.4. Western Blot

Total cellular protein samples were subjected to SDS-PAGE gel (10%, Invitrogen, Waltham, MA, USA) and analyzed with the following antibodies: rabbit polyclonal anti-FOXM1, mouse monoclonal anti-hnRNP C from Santa Cruz Biotechnology (Dallas, TX, USA), or horseradish peroxidase-labeled mouse anti-β-actin antibody from Sigma-Aldrich (St. Louis, MO, USA).

### 2.5. RT-PCR and RT-qPCR

One microgram total RNA was treated with DNase I (Thermo fisher scientific, Waltham, MA, USA), and followed by reverse transcription with random primers (hexadeoxynucleotides) and Moloney Murine Leukemia Virus (MMLV) reverse transcriptase (Promega, Madison, WI, USA) in a total 20 μL reaction system. Then, one microliter of the cDNA was used in PCR amplification with Taq DNA polymerase (Invitrogen, Waltham, MA, USA) and the following primers: 5’-GGAAGATGAAGCCACTGCTACCACG-3’ and 5’-CAAGGGAGGGCTCTCCACTTTGATG-3’ for FOXM1 exon 9 alternative splicing, 5′-GCTACTTGACATTGGACCAGGTG-3′ and 5’- TCTCCTCTTTCCCTGGTCCTGC-3’ for FOXM1 exon 6 and 9 alternative splicing, 5′-GAAGGTGAAGGTCGGAGTC-3′ and 5′-GAAGATGGTGATGGGATTTC-3′ for internal control GAPDH. FOXM1 isoforms generated by minigene were analyzed by using 5′-TGGGGAACAGGTGGTGTTTGG-3′ and 5′-GCTCCTCGCCCTTGCTCACCA-3′ for FOXM1a, 5’-GGAAGATGAAGCCACTGCTACCACG-3’ and 5′-GCTCCTCGCCCTTGCTCACCA-3′ for FOXM1b/c. Quantitative PCR (qPCR) was performed using ChamQ Universal SYBR qPCR Master Mix (Vazyme Biotech, Nanjing, China) in the CFX96 qPCR detection system (Bio-Rad, Hercules, CA, USA) with following primers: 5’-CGGCTGCCCTACCTACGGA-3’ and 5’-GGGAGGGCAGCTATTAGGA-3’ for PLK1, 5’-GGGCAAGTTCAGCAACATCGTGGA-3’ and 5’-GTAGCCGCCTTTCAGGATATACATC-3’ for CDC25B, 5′-AATAAGGCGAAGATCAACATGGC-3′ and 5′-TTTGTTACCAATGTCCCCAAGAG-3′ for CCNB1, 5′-CCCAGCACAATGAAGATCAA-3′ and 5′-ACATCTGCTGGAAGGTGGAC-3′ for β-actin. The relative expression levels of target genes were calculated by using the 2^−∆∆Ct^ method. 

### 2.6. Colony Formation Assays

One thousand cells (per well) were seeded to 6-well plates in triplicate for each biological repeat and cultured for 10 days. The cells were fixed in paraformaldehyde and stained with crystal violet. Colonies containing >50 cells were counted.

### 2.7. Tumor Induction in Nude Mice

CAL 27 cells stably transfected with FOXM1a, FOXM1c, or vector plasmid were implanted by dorsal subcutaneous inoculation at both sides of Balb/c nude mice (1 × 10^6^ cells per side, 5 or 4 mice per group). Tumor volumes were measured every two or three days. The volume of tumors was calculated as (length × width^2^)/2. Mice were sacrificed on Day 22. The tumors were dissected out and weighed. 

### 2.8. RNA Pulldown

Biotin-labeled FOXM1 RNA oligonucleotides wt10 (5′ Biotin-AAUUUUAUCUUUCUUUGTT 3′ wild type exon 9 exonic splicing suppressor), mt 10 (5′ Biotin-AAUCUCAACCUACUUUGTT 3′ mutant exon 9 exonic splicing suppressor, the mutation was underlined), and C+ (5′ Biotin-UUGUUUUUCUCAACACCUCCTT 3′ positive control sequence of hnRNP C binding) were synthesized by Takara (Dalian, China). The total cellular extract of 293 cells was prepared using radioimmunoprecipitation assay (RIPA) buffer (Thermo fisher scientific, Waltham, MA, USA). One microliter biotin-labeled RNA oligonucleotide (20 μM) was immobilized onto 10 μL of NeutrAvidin beads (Pierce, Appleton, WI, USA). The RNA-bead complexes were then incubated with 100 μL of 293 total cell extract diluted in 300 μL 1× binding buffer (20 mM Tris, 200 mM NaCl, 6 mM EDTA, 5 mM potassium fluoride, 5 mM β-glycerophosphate, 2 μg/mL aprotinin, pH 7.5) at 4 °C for 2 h. After washing for three times with 1× binding buffer, the bound proteins were eluted by SDS (sodium dodecyl sulfate) sample buffer and analyzed by Western blot. 

### 2.9. TCGA Data Analysis

The expression levels of FOXM1 and hnRNP C in The Cancer Genome Atlas Program (TCGA) head and neck squamous cell carcinoma were obtained from OncoDB database [23] and analyzed by Graphpad 8.0. The correlation between FOXM1 and hnRNP C expression levels were analyzed in Timer2.0 online database [24].

### 2.10. Statistical Analysis

The one-way analysis of variance (ANOVA) test was used to analyze the differences between the means. The difference between the means of two independent groups was analyzed by *t* test. The Spearman correlation between FOXM1 and hnRNP C was analyzed by Graphpad Prism 8.0. 

## 3. Results

### 3.1. FOXM1a Inhibits Cell Proliferation and Colony Formation

**Figure 1:**
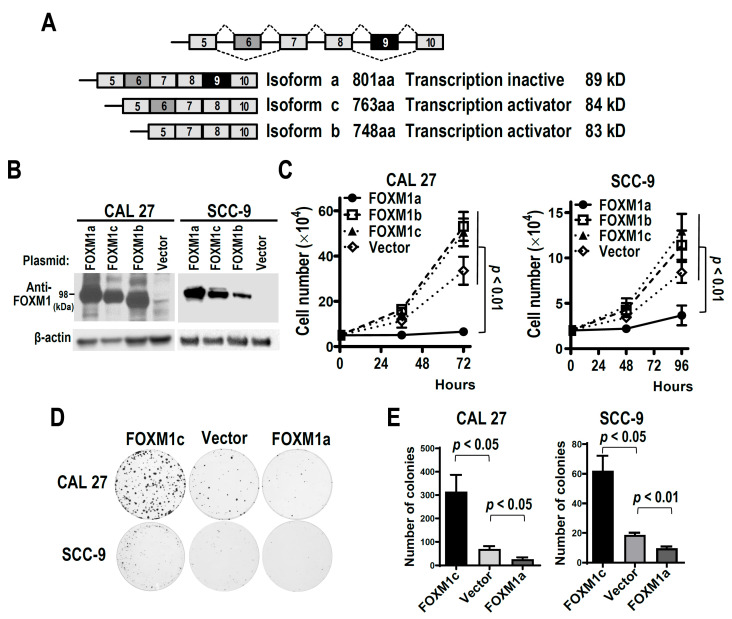
FOXM1a inhibits cancer cell proliferation. (**A**) Schematic diagram of the alternative splicing of FOXM1 exon 6 and 9. FOXM1a with both exon 6 and 9 encodes full length FOXM1. Isoform c without exon 9 encodes FOXM1c protein. Isoform b without both exon 6 and 9 encodes FOXM1b protein. (**B**) Western blot showed exogenous expression of T7 tagged FOXM1a, FOXM1b, or FOXM1c in CAL 27 or SCC-9 cells. β-actin served as loading control. (**C**) Growth curve of CAL 27 or SCC-9 cells stably transfected with T7 tagged FOXM1a, FOXM1b, FOXM1c expression plasmid or vector control plasmid (*n* = 3). (**D**,**E**) Colony formation of CAL 27 or SCC-9 cells stably transfected with T7 tagged FOXM1a, FOXM1b, and FOXM1C (*n* = 3). Representative colony images are shown in panel D. The original image of Figure 1B can be found in Supplementary File S1.

*FOXM1* is a well-known oncogene and is involved in cell proliferation and cell cycle progress. FOXM1 gene contains 10 exons. FOXM1 has at least four isoforms generated by alternative splicing of exon 6 and 9 (Figure 1A). FOXM1a contains exon 9 and is transcriptionally inactive. Both FOXM1c and FOXM1b lose exon 9 and are transcription activators. FOXM1c and FOXM1b have been reported as oncogenes. However, the function of FOXM1a in tumorigenesis remains unclear. In OSCC cells, FOXM1c is the most abundant isoform, and the expression levels of FOXM1a are much lower than of FOXM1c (Figure S1). We established stable expressions of FOXM1a, FOXM1b, FOXM1c, or vector control in two OSCC cell lines: CAL 27 or SCC-9 (Figure 1B). Overexpression of FOXM1a significantly inhibited cell proliferation in both cell lines compared with vector transfection control. In contrast, overexpression of FOXM1b or FOXM1c promoted cell growth (Figure 1C). Moreover, FOXM1a overexpression also significantly inhibited colony formation in both cell lines, whereas FOXM1c overexpression significantly promoted colony formation in CAL 27 and SCC-9 cells (Figure 1D,E). These results suggest that FOXM1a plays negative roles in cancer cell proliferation. 

We also overexpressed FOXM1a and FOXM1c in HEK 293 cells. Similarly, FOXM1a overexpression suppressed the cell proliferation of HEK 293 cells (Figure S2A), indicating that FOXM1a may also have negative effect on the cell proliferation of immortalized cells.

### 3.2. FOXM1a Suppresses Tumor Formation In Vivo

**Figure 2:**
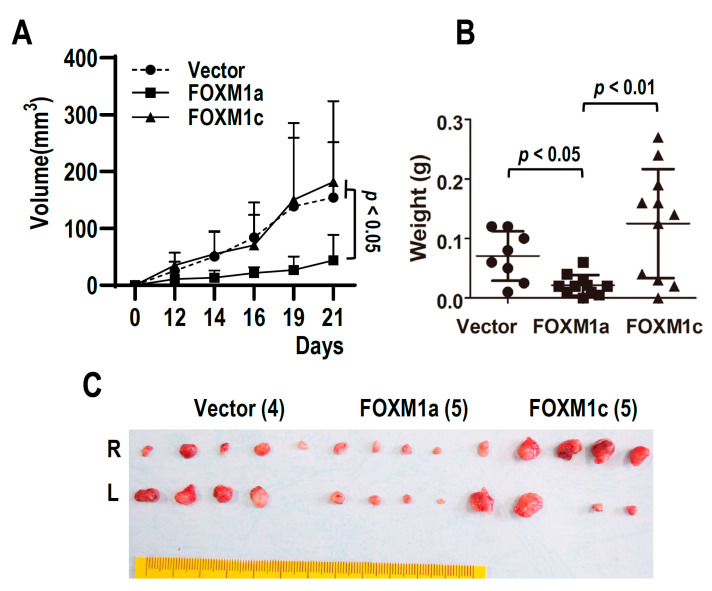
FOXM1a is a tumor suppressor. (**A**) CAL 27 cells stably transfected with FOXM1a, FOXM1c, or vector plasmid were subcutaneously inoculated into nude mice (*n* = 4 or 5). Tumor formation was checked every two or three days. Note: the measured tumor sizes included the thickness of mouse skin in vivo. (**B**,**C**) Tumors were dissected out (L, left; R, right) and weighed on Day 22.

To investigate the roles of FOXM1a in tumorigenesis in vivo, we inoculated CAL 27 stably transfected with FOXM1a, FOXM1c, or empty vector into nude mice. Cells with FOXM1a overexpression showed significantly slower tumor formation than those with FOXM1c overexpression or control vector (Figure 2A). FOXM1a overexpression also significantly reduced tumor weight (Figure 2B,C), indicating that FOXM1a is a tumor suppressor.

### 3.3. FOXM1a Suppresses the Expression of CDC25B, PLK1, and CCNB1

**Figure 3:**
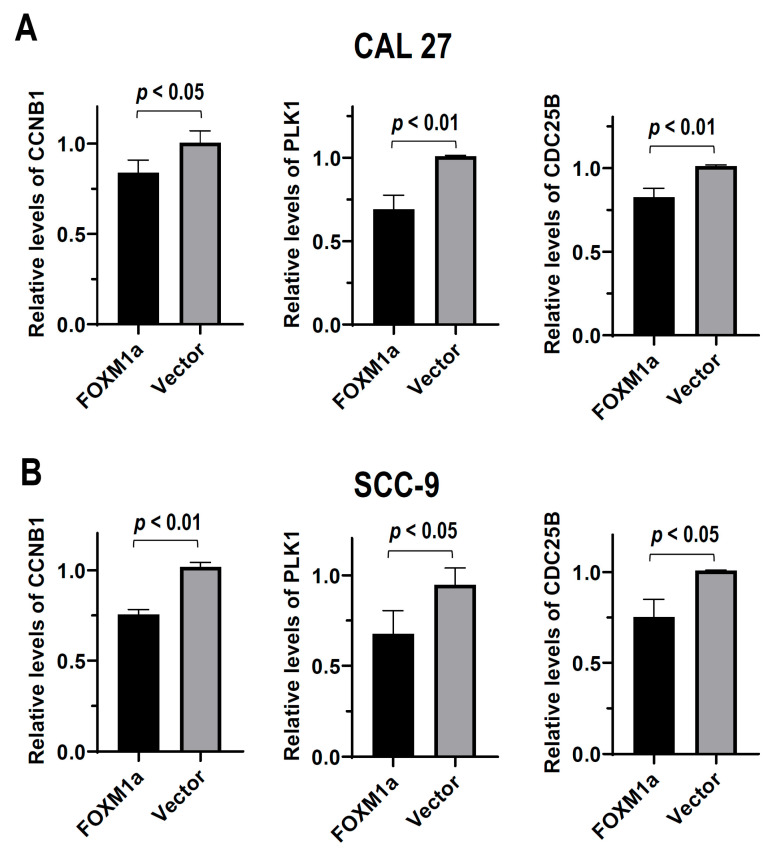
FOXM1a suppresses the expression of CCNB1, PLK1, and CDC25B. The expression levels of CCNB1, PLK1, and CDC25B in CAL 27 (**A**) or SCC-9 (**B**) cells transfected with T7 tagged FOXM1a expression plasmid or vector control plasmid (*n* = 3) were analyzed by RT-qPCR.

To understand the regulatory mechanism of FOXM1a in cancer cell proliferation and tumorigenesis, we analyzed the effects of FOXM1a on the expression of FOXM1c target genes, including *CDC25B*, *PLK1*, and *CCNB1*, which are essential for cell proliferation. We found that FOXM1a overexpression significantly decreased the expression of CDC25B, PLK1, and CCNB1 in both CAL 27 and SCC-9 cells (Figure 3), indicating that FOXM1a may inhibit cell proliferation and tumor formation through downregulating the expression of these key genes. 

### 3.4. HnRNP C Binds to an Exonic Splicing Suppressor in FOXM1 Exon 9

**Figure 4:**
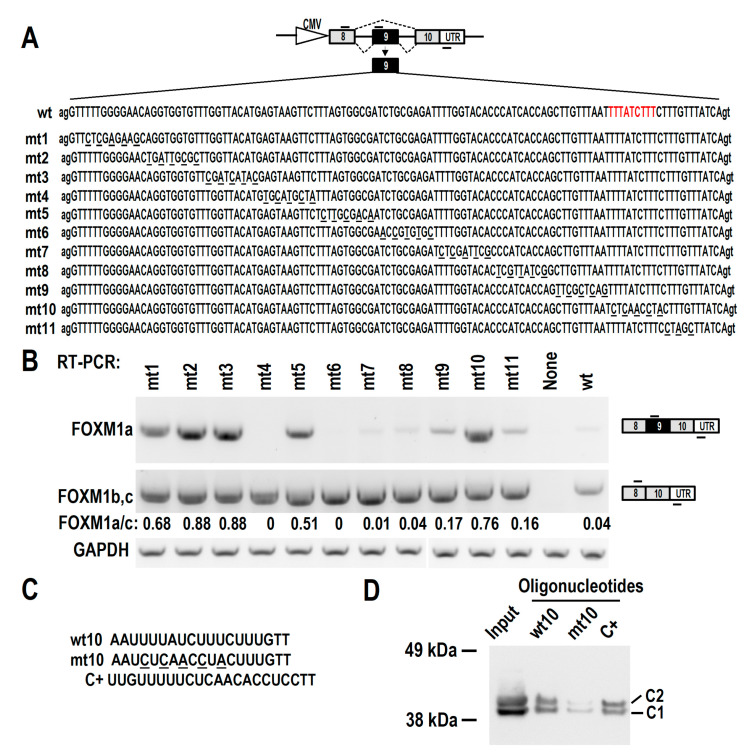
HnRNP C interacts with an ESS (exonic splicing suppressor) motif in FOXM1 exon 9. (**A**) Schematic diagram of FOXM1 minigene. The genomic sequence from 3′ part of exon 8 to 5′ part of exon 10 of FOXM1 was amplified from CAL 27 cells and driven by CMV promoter in pEGFP-N1 vector. To map potential regulatory motifs, exon 9 in minigene was serially mutated. (**B**) Wild-type (wt) or mutated (mt) minigenes were transfected into 293 cells. Alternative splicing of exon 9 was analyzed by RT-PCR. FOXM1 a/c represents the ratio of band intensities of FOXM1a vs. FOXM1b, c. Diagrams on the right show the structures of spliced products and primer positions (short lines above or below exons). (**C**,**D**) The interaction between ESS and hnRNP C was analyzed by using an RNA pulldown assay. Oligonucleotide RNAs, including wild type (wt10) or mutant (mt10) ESS, and a positive control sequence (C+) were biotinylated and incubated with 293 total cellular extract. RNA-interacting proteins were separated in SDS-PAGE gel and blotted with a mouse anti-hnRNP C antibody. HnRNP C has two isoforms, C1 and C2, generated by alternative splicing. In panel A, the corresponding position of wt10 in exon 9 is highlighted with red color. Beads: RNA-interacting proteins. The original image of Figure 4B,D can be found in Supplementary File S1.

Heterogeneous nuclear ribonucleoprotein (HnRNP) proteins often bind exonic splicing suppressor (ESS) and inhibit exon inclusion. By serial mutation of the exon 9 sequence, we screened whole exon 9 and found three motifs were responsible for exon 9 skipping, including the corresponding wild-type sequences of mt2, mt3, mt10, and mt5 (ordered by the ratio of FOXM1a vs. FOXM1b/c in Figure 4A,B). Among these motifs, the wt10 sequence shows a characteristic of U-rich (UUUAUCUUU) and may be an exonic splicing suppressor (ESS). Because hnRNP C, a heterogeneous nuclear ribonucleoprotein protein, was reported to recognize U-rich motifs [25], we hypothesized that hnRNP C may interact with this ESS and repress exon 9 inclusion (Figure 4C). Therefore, we performed an RNA pulldown assay to analyze the interaction between this ESS motif and hnRNP C proteins. As expected, hnRNP C can bind to its known RNA motif (C+). Importantly, wt10 RNA showed specific binding to hnRNP C proteins. Mutation of wt10 (mt10) significantly reduced hnRNP C binding (Figure 4D). These data suggest that hnRNP C interacts with the wt10 ESS motif and may inhibit exon 9 inclusion.

Interestingly, mutation mt4 and mt6 almost completely abolished the inclusion of exon 9 compared with the wild-type control (Figure 4B), suggesting that there are potential exonic splicing enhancers (ESEs) in these regions. The wild-type sequences of mt4 and mt6 are purine rich sequences. Further studies are required for exploring the regulatory mechanisms of these potential ESEs.

### 3.5. HnRNP C Suppresses the Inclusion of FOXM1 Exon 9 and Is Required for OSCC Cell Proliferation

**Figure 5:**
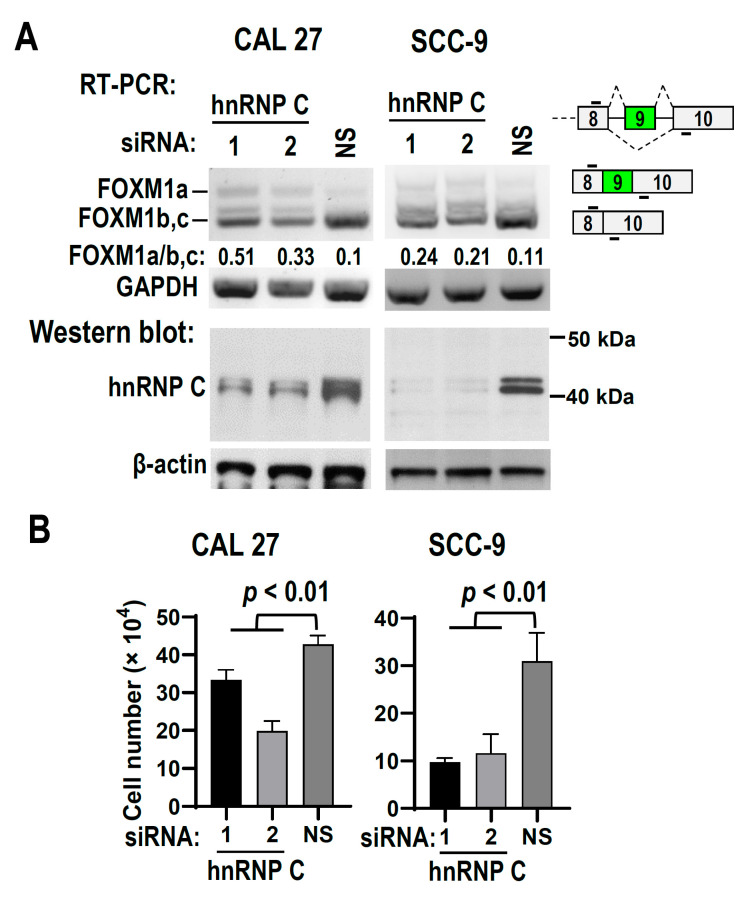
HnRNP C suppresses the inclusion of FOXM1 exon 9 and is required for OSCC cell proliferation. (**A**) Knockdown of hnRNP C promotes inclusion of FOXM1 exon 9. Total RNA and protein were collected from CAL 27 or SCC-9 cells transfected by anti-hnRNP C or NS siRNA. Alternative splicing of FOXM1 exon 9 was analyzed by RT-PCR. Diagrams on the right show the structures of spliced products and primer positions (short lines above or below exons). GAPDH serves as a loading control. Knockdown efficiency of hnRNP C was analyzed by Western blotting. β-actin served as a loading control. (**B**) Knockdown of hnRNP C represses OSCC proliferation. CAL 27 or SCC-9 cells were transfected with 20 nM hnRNP C siRNAs or NS siRNA twice in a 48 h interval, respectively. Cell numbers were counted at 96 h after the first transfection. The original image of Figure 5A can be found in Supplementary File S1.

Next, we explored the roles of hnRNP C in the alternative splicing of FOXM1 exon 9. HnRNP C expression was silenced with two different anti-hnRNP C siRNAs in both CAL 27 and SCC-9 cells. CAL 27 cells with hnRNP C knockdown showed significantly increased levels of FOXM1a and exon 9 inclusion compared with those cells treated with control siRNA (the ratio of FOXM1a/b,c increased from 0.1 to 0.33 and 0.51). Similarly, SCC-9 cells with hnRNP C knockdown also showed significantly increased levels of FOXM1a and exon 9 inclusion compared with those cells treated with control siRNA (the ratio of FOXM1a/b,c increased from 0.11 to 0.21 and 0.24) (Figure 5A), indicating that hnRNP C may bind to ESS motif of exon 9 and suppress its inclusion. In line with the tumor suppressive function of FOXM1a, knockdown of hnRNP C significantly suppressed cell proliferation in both CAL 27 and SCC-9 cells (Figure 5B), indicating that hnRNP C is required for OSCC cell proliferation. These results were also further confirmed in HEK 293 cells (Figure S2B,C).

### 3.6. HnRNP C Is Co-Expressed with FOXM1 in Cancers

**Figure 6:**
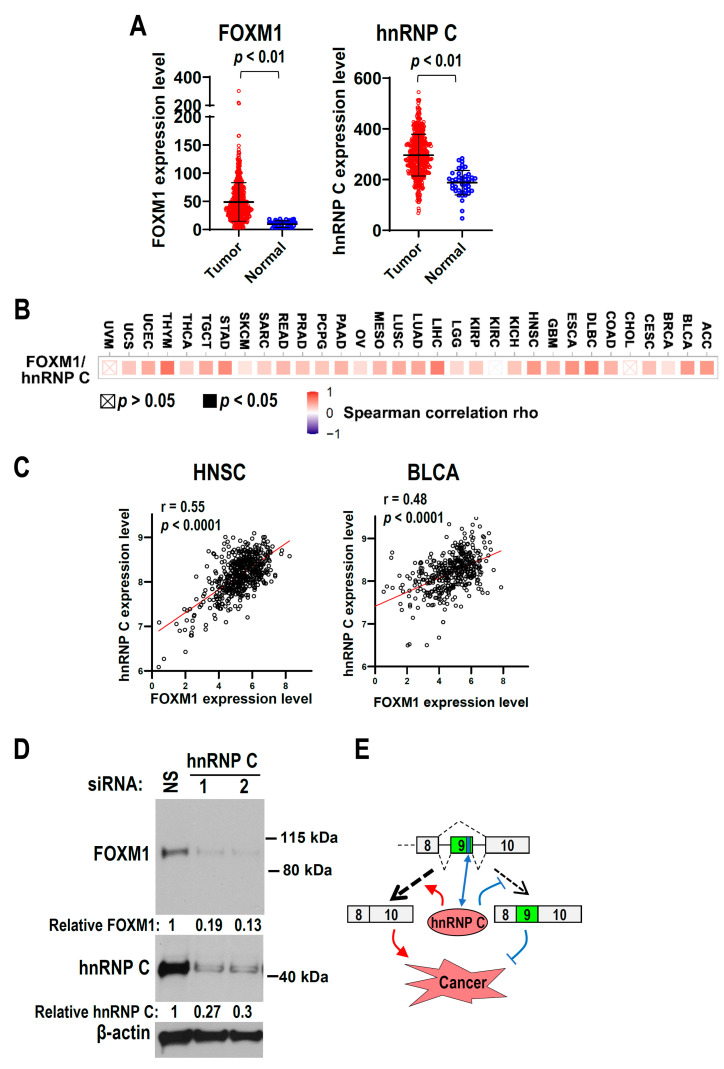
HnRNP C is positively co-expressed with FOXM1 in cancers. (**A**) Expression levels of FOXM1 or hnRNP C (encoded by *HNRNPC* gene) in TCGA head and neck squamous cell carcinoma (HNSC) or normal tissues. (**B**) The correlations between FOXM1 and hnRNP C expression levels in TCGA cancers were analyzed in Timer2.0 online database. (**C**) The scatter plots show the correlations between FOXM1 and hnRNP C expression levels in HNSC or bladder carcinoma (BLCA). (**D**) The expression of FOXM1 protein was analyzed by Western blotting in CAL 27 cells with hnRNP C knockdown. (**E**) A model of the novel regulatory pathway of hnRNP C/FOXM1. FOXM1 exon 9 inclusion and FOXM1a expression are suppressed by hnRNP C, which promotes tumorigenesis. The original image of Figure 6D can be found in Supplementary File S1.

FOXM1 is an oncogene and is often overexpressed in multiple cancers. We analyzed the expression of FOXM1 and hnRNP C in TCGA head and neck squamous cell carcinoma (HNSC) and found that FOXM1 was significantly up-regulated in tumor tissues compared with normal controls (Figure 6A). Interestingly, hnRNP C is also significantly up-regulated in HNSC tissues (Figure 6A). Moreover, FOXM1 expression levels are significantly and positively correlated with hnRNP C expression levels in most TCGA cancer types (29 out of 32). The correlation between FOXM1 and hnRNP C expression is strong (r = 0.55) in HNSC (Figure 6B,C). Another example is bladder carcinoma (BLCA), which showed a relatively strong correlation (r = 0.48) between FOXM1 and hnRNP C expression (Figure 6C). These results confirmed that increased FOXM1 expression is significantly positively correlated with hnRNP C expression in cancers. Moreover, knockdown of hnRNP C reduced the expression of FOXM1 protein (Figure 6D), which is correlated with the positive co-expression of FOXM1 and hnRNP C in cancers. 

## 4. Discussion

*FOXM1* is an oncogene and is often overexpressed in cancers [26]. FOXM1 expression is required for cell cycle progression and cell proliferation. FOXM1 is essential for G1/S transition, mitosis entry, and execution [27]. Moreover, FOXM1 plays important roles in other aspects of tumorigenesis. FOXM1 promotes metastasis by inducing epithelial–mesenchymal transition [28]. Overexpression of FOXM1 enhanced tumor cell invasion, migration, and angiogenesis [29,30]. FOXM1 total expression was dramatically upregulated in pancreatic cancer, however, FOXM1a expression did not increase significantly [31]. Klinhom-on et al. showed that normal adjacent tissues of cholangiocarcinoma only expressed the FOXM1a isoform, whereas the FOXM1c and FOXM1b isoforms were expressed only in tumor tissues of cholangiocarcinoma [32].

The expression of FOXM1 is tightly regulated in the transcriptional, post-transcriptional, and post-translational levels. Transcriptional regulation of FOXM1 has been extensively studied. For example, several transcriptional factors, including signal transducer and activator of transcription 3 (STAT3) and cAMP responsive element-binding protein (CREB), have been shown to interact with FOXM1 promoter and upregulate its expression [33]. For post-transcriptional regulation of FOXM1 expression, a number of microRNAs can downregulate FOXM1 expression by binding to 3′ UTR of its mRNA [33]. However, the regulatory mechanism of pre-mRNA alternative splicing of FOXM1 remains largely unclear. In the present study, we screened the whole exon 9 and identified a key exonic splicing suppressor, which is responsible for the skipping of exon 9. Moreover, we also uncovered three more motifs, which may also suppress exon 9 inclusion. 

In contrast to FOXM1b and FOXM1c, FOXM1a is transcriptionally inactive in the expression of their many target genes. But FOXM1a can drive the expression of some distinct genes. Interestingly, Barger et al. found that transcriptional targets of FOXM1a are obviously different from FOXM1b and FOXM1c [34]. Interestingly, these FOXM1a-specific target genes are highly enriched in the innate-immunity-associated pathway, including DExD/H-box helicase 60 (DDX60), ISG15 ubiquitin like modifier (ISG15), interferon alpha inducible protein 6 (IFI6), interferon induced protein with tetratricopeptide repeats 1 (IFIT1), interferon induced transmembrane protein 1 (IFITM1), interferon induced protein with tetratricopeptide repeats 3 (IFIT3), and sterile alpha motif domain containing 9 (SAMD9), suggesting that FOXM1a may be involved in immune responses. In contrast, the target genes of both FOXM1b and FOXM1c are enriched in the cell cycle. An example is KIF4A, which plays essential functions in the anaphase of mitosis and cytokinesis [35]. KIF4A expression is transactivated by FOXM1b and FOXM1c, not FOXM1a [36]. Slight ectopic expression of FOXM1c or FOXM1b, not FOXM1a, promoted pancreatic tumor growth and metastasis [31]. 

The FOXM1 protein contains three domains, a DNA binding domain, an N-terminal domain, and a C-terminal transactivation domain. Interestingly, the N-terminal 232 residues of FOXM1 play negative roles in its transcription activity [37]. Exon 9 encodes a fragment of 38 amino acids in the C-terminal transactivation domain. The fragment encoded by exon 9 may interfere with the role of the C-terminal transactivation domain and result in no transactivational activity by FOXM1a. Since FOXM1a contains the DNA-binding domain, it may compete for binding to FOXM1-binding sites with FOXM1b and FOXM1c and act as a dominant-negative isoform of FOXM1. However, the role of FOXM1a in tumorigenesis remains largely unclear. In our study, we discovered that overexpression of FOXM1a in OSCC cells significantly impaired their tumorigenesis in vivo, which suggests that full-length isoform FOXM1a is a tumor suppressor. In addition, the regulatory mechanisms of FOXM1 exon 9 alternative splicing are also unclear. By serial mutation of exon 9, we identified a key regulatory motif for exon 9 inclusion, and hnRNP C was the corresponding key regulator. 

HnRNP C, a member of heterogeneous nuclear ribonucleoproteins (hnRNP) splicing factor family, is abundantly expressed in the nucleus and is tightly associated with nascent transcripts [38,39]. In the present study, we found that hnRNP C inhibited the inclusion of FOXM1 exon 9. As an important splicing factor, hnRNP C plays key roles in the regulation of alternative RNA splicing [40]. Moreover, hnRNP C is also involved in many other cellular functions, such as identification of m6A modification [41], mRNA stability [42], translation efficiency [43], measuring RNA length and determination of the nuclear export of transcripts [44], and selection of alternative polyadenylation sites [45]. Deletion of hnRNP C in embryonic mouse is lethal [46]. Notably, hnRNP C is upregulated and associated with poor prognosis in some cancers [47]. HnRNP C can promote tumorigenesis by enhancing the IRES-dependent translation of oncogene c-Myc [43], promoting ZEB1 expression [48], increasing pro-metastatic isoform expression of TAF8 gene in an m6A-dependent manner [49], and so on. However, the mechanisms of hnRNP C oncogenic functions need to be further explored, especially in the targets of alternative splicing. We found hnRNP C knockdown significantly reduced OSCC proliferation, which may be attributed to the increased inclusion of FOXM1 exon 9 and expression of the tumor-suppressive FOXM1a isoform. Our finding suggests that blocking the interaction between hnRNP C and the ESS in exon 9 may be a novel anticancer method.

HnRNP C regulates pre-mRNA alternative splicing by binding to uridine-rich motifs in introns or exons via its amino-terminal RNA recognition motif (RRM, aa 16–87). A large scale of iCLIP assay revealed that hnRNP C tetramer binds to precisely spaced uridine tracts in intron and results in exon skipping [40]. Nasrin et al. showed that hnRNP C bound to an ESS motif (four uridines) and suppressed exon 10 inclusion of MUSK pre-mRNA [50]. HnRNP C is also able to bind contiguous uridines in IRES site of Unr mature mRNA [51]. In line with these findings, we found that hnRNP C bound to a new uridine rich ESS motif (exonic splicing suppressor, UUUUAUCUUU) in FOXM1 exon 9 and suppressed its inclusion. 

Dysregulated alternative splicing of pre-mRNA has been increasingly associated with tumorigenesis [52]. Previously, we found that HNRNPK, a member of heterogeneous nuclear ribonucleoproteins, promoted exon 4 inclusion of SPIN1, an oncogenic histone code reader, to increase the expression of oncogene CCND1 in OSCC cells [53]. HNRNPA1, another member of heterogeneous nuclear ribonucleoproteins, is required for the inclusion of CDK2 exon 5 in OSCC cells [54]. Huang et al. found that hnRNP C promoted OSCC proliferation, migration, and EMT [55]. HnRNP C is also a member of heterogeneous nuclear ribonucleoproteins and is abundant in cells. In the present study, we further uncovered that hnRNP C may be important for cancer cell proliferation due to its inhibition on the expression of tumor suppressive FOXM1a isoform in OSCC cells. Therefore, we demonstrated that hnRNP C played roles in inhibiting the expression of the tumor suppressive isoform FOXM1a of oncogene FOXM1. 

## 5. Conclusions

In conclusion, we proposed a model of the novel regulatory pathway of hnRNP C/FOXM1, namely, FOXM1 exon 9 inclusion and FOXM1a expression are suppressed by the interaction between hnRNP C and an exonic splicing suppressor in exon 9, and this promotes tumorigenesis in OSCC (Figure 6E). Our findings may help design the novel anticancer method by blocking the interaction between hnRNP C and the ESS in exon 9. 

## Figures and Tables

**Table 1:** Primers used for serial mutation of human FOXM1 exon 9 splicing minigene.

Mutation	Forward Primer	Backward Primer
mt1	TATTTCCATAGGTTCTCGAGAAGCAGGTGGTGTTTGGT	ACCAAACACCACCTGCTTCTCGAGAACCTATGGAAATA
mt2	AGGTTTTTGGGGAACTGATTGCGCTTGGTTACATGAGTA	TACTCATGTAACCAAGCGCAATCAGTTCCCCAAAAACCT
mt3	GGAACAGGTGGTGTTCGATCATACGAGTAAGTTCTTTAG	CTAAAGAACTTACTCGTATGATCGAACACCACCTGTTCC
mt4	GTGTTTGGTTACATGTGCATGCTATTTAGTGGCGATCTGC	GCAGATCGCCACTAAATAGCATGCACATGTAACCAAACAC
mt5	TACATGAGTAAGTTCTCTTGCGACAATCTGCGAGATTTTG	CAAAATCTCGCAGATTGTCGCAAGAGAACTTACTCATGTA
mt6	GTTCTTTAGTGGCGAACCGTGTGCTTTTGGTACACCCATCA	TGATGGGTGTACCAAAAGCACACGGTTCGCCACTAAAGAAC
mt7	GGCGATCTGCGAGATCTCGATTCGCCCATCACCAGCTTGTT	AACAAGCTGGTGATGGGCGAATCGAGATCTCGCAGATCGCC
mt8	GAGATTTTGGTACACTCGTTATCGGCTTGTTTAATTTTATC	GATAAAATTAAACAAGCCGATAACGAGTGTACCAAAATCTC
mt9	TACACCCATCACCAGTTCGCTCAGTTTTATCTTTCTTTGTT	AACAAAGAAAGATAAAACTGAGCGAACTGGTGATGGGTGTA
mt10	ACCAGCTTGTTTAATCTCAACCTACTTTGTTTATCAGTAA	TTACTGATAAACAAAGTAGGTTGAGATTAAACAAGCTGGT
mt11	GTTTAATTTTATCTTTCCTAGCTTATCAGTAAGTCTGAGC	GCTCAGACTTACTGATAAGCTAGGAAAGATAAAATTAAAC

## Data Availability

All relevant data are within the paper.

## Supplementary Materials

The following supporting information can be downloaded at: https://www.mdpi.com/article/10.3390/biom13091331/s1, File S1: Original images of RT-PCR and Western blot.
