# Supplementary material for: FOXM1a Isoform of Oncogene FOXM1 Is a Tumor Suppressor Suppressed by hnRNP C in Oral Squamous Cell Carcinoma
Source: Biomolecules. 2023 Aug 30;13(9):1331. doi: 10.3390/biom13091331 (PMC10526205; doi:10.3390/biom13091331)
Supplement: Supplementary file 1 [file biomolecules-13-01331-s001.zip › Figure S2.pdf]

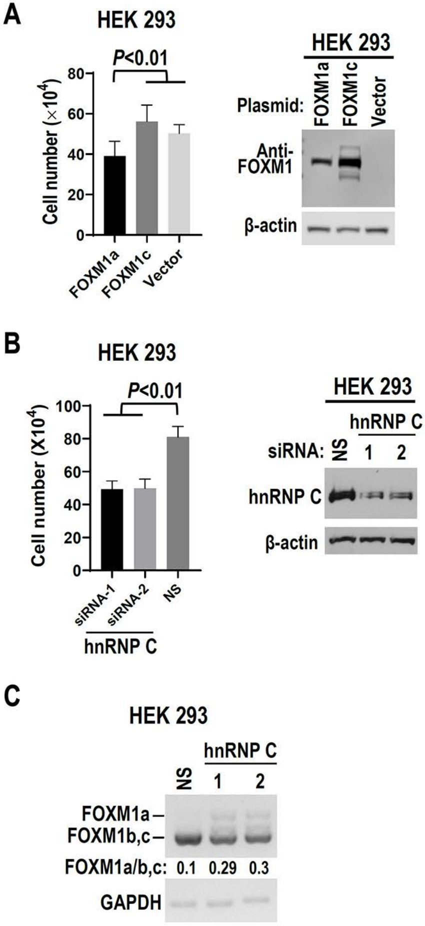

Figure S2. Effects of FOXM1a and hnRNP C on HEK 293 proliferation. (**A**) HEK 293 cells were stably transfected with T7 tagged FOXM1a, FOXM1c expression plasmid or vector control plasmid (n=3). Cell number was counted at four days after inoculation. Overexpression of FOXM1a or FOXM1c was confirmed by Western blotting. (**B**) Knockdown of hnRNP C represses HEK 293 cell proliferation. HEK 293 were transfected with 20 nM hnRNP C siRNAs or NS siRNA twice in a 48-hour interval, respectively. Cell number was counted at four days after the first transfection. Knockdown of hnRNP C was confirmed by Western blotting. (**C**) Alternative splicing of FOXM1 exon 9 was analyzed by RT-PCR in HEK 293 cells in Figure S2B.
