# Supplementary figures and images for: FOXM1a Isoform of Oncogene FOXM1 Is a Tumor Suppressor Suppressed by hnRNP C in Oral Squamous Cell Carcinoma
Source: Biomolecules. 2023 Aug 30;13(9):1331. doi: 10.3390/biom13091331 (PMC10526205; doi:10.3390/biom13091331)

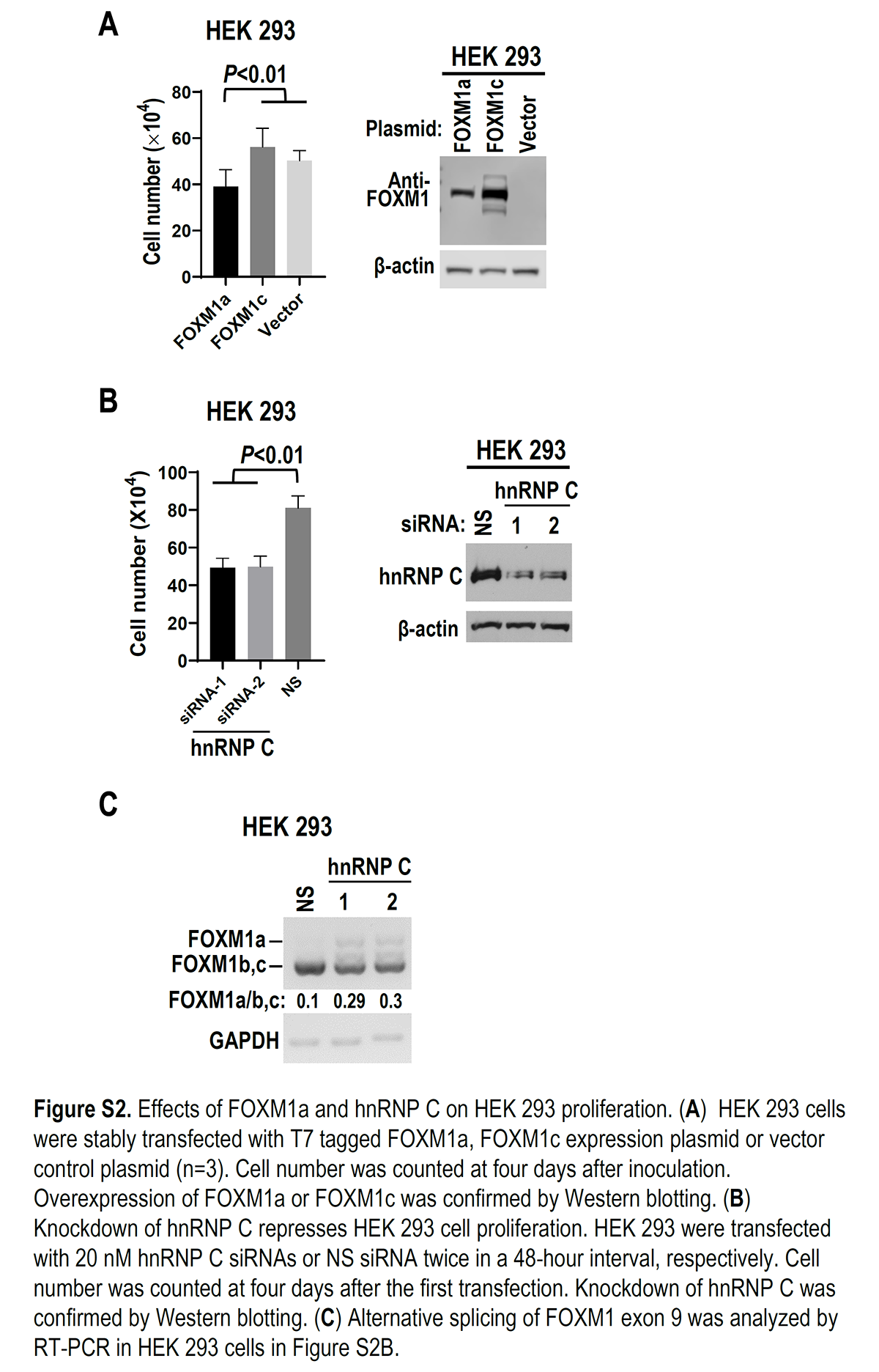

Supplement: Supplementary file 1 [file biomolecules-13-01331-s001.zip › Figure S2.tif]
